# Etanercept and venous thromboembolism: a case series

**DOI:** 10.1186/1752-1947-4-12

**Published:** 2010-01-15

**Authors:** Ashima Makol, Madhusudan Grover, Carla Guggenheim, Houria Hassouna

**Affiliations:** 1 Internal Medicine, Michigan State University, East Lansing, MI, USA; 2 Internal Medicine and Rheumatology, Michigan State University, East Lansing, MI, USA; 3 Department of Medicine, Special Coagulation Laboratory, Michigan State University, East Lansing, MI, USA

## Abstract

**Introduction:**

The treatment with antitumor necrosis factor agents has often been associated with the induction of autoantibodies (antinuclear antibodies, anti-double stranded DNA antibodies and antiphospholipid antibodies). The clinical significance of these antibodies remains unclear, but they may predispose to antiphospholipid syndrome with thromboembolic complications. The association of etanercept with thromboembolic events has not been reported previously in the literature.

**Case presentation:**

We describe the cases of three patients with rheumatoid arthritis, psoriatic arthritis and seronegative inflammatory arthritis who were treated with etanercept. They developed deep vein thrombosis and/or pulmonary embolism one to three years after the initiation of etanercept therapy. All three patients had a prolonged activated partial thromboplastin time with a positive lupus anticoagulant that persisted even after 12 weeks.

**Conclusion:**

Although the clinical significance of antiphospholipid antibodies during treatment with antitumor necrosis factor agents remains unclear, they may predispose patients to develop antiphospholipid syndrome when associated with prolonged activated partial thromboplastin time, lupus anticoagulant positivity, or the presence of anti-β2 glycoprotein I. Clinicians must keep this in mind during therapy with antitumor necrosis factor agents in order to prevent, detect and treat potential consequences such as deep vein thrombosis and pulmonary embolism.

## Introduction

Etanercept (Enbrel; Immunex; Thousand Oaks, California) is a dimeric recombinant DNA protein composed of two naturally occurring soluble human tumor necrosis factor (TNF) receptors linked to the Fc portion of IgG1. TNF plays an important role in inflammatory processes and binds into two different types of receptors, wherein one becomes embedded in white blood cells and is responsible for the immune response and the other becomes a soluble TNF receptor used to deactivate TNF and blunt immune response. Etanercept mimics the inhibitory effects of naturally occurring soluble TNF receptors. The only difference is that etanercept is a fusion protein rather than a simple TNF receptor and has a greatly extended half-life in the bloodstream, and therefore has a more profound and long-lasting biologic effect than a naturally occurring soluble TNF receptor.

Pulmonary embolism (PE) has been previously described in the literature with infliximab (monoclonal antibody against TNF) [1]. In this case report, we describe the cases of three patients who developed deep vein thrombosis (DVT) leading to PE during etanercept therapy. All patients were seen at one medical center over a 3-year period.

## Case presentation

We report the cases of three patients with rheumatoid arthritis, psoriatic arthritis and seronegative inflammatory arthritis who were treated with etanercept.

Our first patient is a 60-year-old Hispanic man with a four-year history of rheumatoid arthritis (RA). He was treated previously with a combination of various disease-modifying antirheumatic drugs and corticosteroids. Etanercept therapy, which proved successful in treating his RA, was started for a flare up three years prior to his presentation with right calf swelling and acute onset dyspnea. A spiral computed tomography (CT) scan showed a large filling defect in his right and left main pulmonary arteries. A Doppler scan showed right femoral DVT. He denied any history of smoking, recent immobilization, surgery or travel, or any personal or family history of clotting disorders. He had never been on heparin therapy. His activated partial thromboplastin time (aPTT) was 48 seconds. He tested positive for lupus anticoagulant (LAC) and anticardiolipin antibody (aCL), which persisted for 12 weeks along with prolonged aPTT.

Our second patient is a 56-year-old Caucasian man with psoriatic arthritis who had been treated for three years with once-a-week doses of methotrexate and a daily high dose of prednisone. He had a flare up on that regimen, so etanercept therapy was started. He developed progressively increasing aches in his left calf after one and a half years of therapy. A Doppler scan revealed tibial DVT. He did not smoke and had no previous prolonged immobilization, travel, trauma, or surgery. He had neither a personal nor family history of clotting disorders and he had never been on heparin therapy. His aPTT was found to be 51 seconds and workup was positive for LAC and anti-β2 glycoprotein I. All three remained positive when he returned for follow-up after three months.

Our third patient is a 46-year-old Caucasian woman with seronegative inflammatory arthritis that was poorly controlled by methotrexate for four years. She was started on etanercept and developed left lower extremity swelling and dyspnea after one year of therapy. A scan Doppler showed left tibial DVT. A CT scan of her chest showed left lower lobe PE. This was her first episode of DVT and she denied any recent prolonged immobilization, trauma, surgery or travel, but she had a matrilineal family history of blood clots. The results of a procoagulant workup, including antiphospholipid antibody syndrome (APS) screen, prior to etanercept initiation were negative. She had never been on heparin therapy. Her aPTT was 44 seconds and a workup revealed that she was positive for LAC and aCL. These symptoms remained positive at three months follow-up.

## Discussion

Anti-TNF-alpha agents are a powerful addition to our armamentarium in arthritides therapy. Their role in the pathogenesis of thromboembolic events is complex. The production of autoantibodies (antinuclear antigen, anti-dsDNA and antiphospholipid) during treatment is well documented and there are several mechanisms proposed [2,3], which include the following: 1) TNF-alpha inhibition that causes B-cell activation and autoantibody production through the upregulation of interleukin-10 [3,4]; 2) an increase in Th2 activity [5], and 3) an increase in bacterial infections [6] that leads to the production of antibodies through molecular mimicry.

Antiphospholipids (aPLs) (aCLs, anti-beta2 glycoprotein I or LAC) are found in 2% to 6% of healthy individuals and have been associated with recurrent thromboembolism and fetal loss with APS. However, the induction of antiphospholipids is often considered as a nonspecific marker of activated immune system [7,8] such as cardiac thrombosis (associated with aCLs), and they may disappear over time. Some studies have shown the correlation between treatment with anti-TNF-alpha agents, the appearance of aCLs, and clinical outcomes in terms of disease activity and adverse effects including thromboembolic events [2].

In our patients, the following measurements were obtained before the anticoagulant therapy was started to treat the DVT and/or PE: procoagulant work-up including aPTT with a reference range of 18 to 28 seconds; aPLs with IgM and IgG aCLs measured by enzyme-linked immunosorbent assay (ELISA), levels > 20 MPL/GPL considered positive; anti-beta2 glycoprotein I levels measured by ELISA, > 20 SMU/GMU considered positive; LAC detected using dilute Russell's viper venom assay and confirmed with 1:1 dilution of plasma from patients with normal plasma to rule out factor deficiencies and confirm phospholipid dependency; protein C; protein S; antithrombin III, Factor V Leiden; homocysteine; and prothrombin gene mutation (Table 1). All three patients had prolonged aPTT and tested positive for LAC. The aPLs persisted to be positive at three months follow-up and hence consistent with the diagnosis of APS.

**Table 1:** Procoagulant work-up (Post-clinical event but prior to the initiation of anticoagulant therapy)

	Patient 1	Patient 2	Patient 3
Antinuclear antigen	N	N	N

Anticardiolipin antibodies	P	N	P

Lupus anticoagulant	P	P	P

anti-β2 glycoprotein I	N	P	N

Antithrombin III	N	N	N

Homocysteine	N	N	L

Factor 5 Leiden	N	N	N

Prothrombin mutation	N	N	N

Proteins C and S	N	N	N

N, normal or negative; P, positive; L, low.

One patient had a family history of blood clots but the results of a procoagulant work-up prior to the initiation of etanercept treatment were normal. In addition, these patients were not on any medications that are associated with hypercoagulablity. Although a causal relationship cannot be established, the events had a temporal association with etanercept therapy and hence could be correlated. PE has been described with procainamide-induced lupus and positive aCLs [9]. Also, hypercoagulablity and circulating aCLs has been described with the treatment of infliximab [1].

The data associating anti-TNF agents with hypercoagulablity are still in the preliminary stages, but these cases suggest that etanercept-induced LAC positivity may be a contributing factor to venous thromboembolism. The limitation of this clinical experience, however, is its involvement of only three patients and the unavailability of baseline data on aPLs prior to the initiation of etanercept therapy in two of the three patients. In addition, this clinical observation is insufficient to explain if there is, indeed, an increased risk of thromboembolic events associated with etanercept in these patients as compared with the general population.

## Conclusion

Our observations have a potentially strong impact because anti-TNF agents are widely used in a variety of conditions across several aspects of medical practice. Although the role of aPLs in the development of thromboembolism in our patients is uncertain, the potential complications of anti-TNF therapy should be remembered in order to prevent, detect, and manage thrombotic events.

## Abbreviations

aCLs: anticardiolipin antibody; ANA: antinuclear antigen; aPLs: antiphospholipid antibodies; APS: antiphospholipid syndrome; CT: computed tomography; DVT: deep vein thrombosis; ELISA: enzyme-linked immunosorbent assay; LAC: lupus anticoagulant; PE: pulmonary embolism; RA: rheumatoid arthritis; TNF: tumor necrosis factor.

## Consent

Written informed consent was obtained from the patients for publication of these case reports and any accompanying images. Copies of the written consent are available for review by the Editor-in-Chief of this journal.

## Competing interests

The authors declare that they have no competing interests.

## Authors' contributions

AM and MG collected clinical data, reviewed the literature on the topic, and drafted the manuscript. CG and HH reviewed and critically revised the manuscript. All authors read and approved the final manuscript.
